# Amyloid Beta_1–42_ and the Phoshorylated Tau Threonine 231 in Brains of Aged Cynomolgus Monkeys (*Macaca fascicularis*)

**DOI:** 10.3389/fnagi.2014.00313

**Published:** 2014-11-10

**Authors:** Huda Shalahudin Darusman, Albert Gjedde, Dondin Sajuthi, Steven J. Schapiro, Otto Kalliokoski, Yuli P. Kristianingrum, Ekowati Handaryani, Jann Hau

**Affiliations:** ^1^Department of Experimental Medicine, Faculty of Health Science, University of Copenhagen, Copenhagen, Denmark; ^2^Department of Anatomy, Physiology and Pharmacology, Faculty of Veterinary Medicine, Bogor Agricultural University, Bogor, Indonesia; ^3^Department of Neuroscience and Pharmacology, Faculty of Health Science, University of Copenhagen, Copenhagen, Denmark; ^4^Center for Functionally Integrative Neuroscience, University of Aarhus, Aarhus, Denmark; ^5^Department of Radiology and Radiological Science, Johns Hopkins University, Baltimore, MD, USA; ^6^Department of Neurology and Neurosurgery, McGill University, Montréal, QC, Canada; ^7^Primate Research Center, Bogor Agricultural University, Bogor, Indonesia; ^8^Department of Veterinary Sciences, The University of Texas MD Anderson Cancer Center, Bastrop, TX, USA; ^9^Department of Pathology, Faculty of Veterinary Medicine, University of Gajah Mada, Yogyakarta, Indonesia; ^10^Division of Pathology, Department of Clinic, Reproduction and Pathology, Faculty of Veterinary Medicine, Bogor Agricultural University, Bogor, Indonesia

**Keywords:** degenerative disease, immunohistochemistry, senile plaques, neurofibrillary tangles, cerebral amyloid angiopathy

## Abstract

Pathological hallmarks indicative of Alzheimer’s disease (AD), which are the plaques of amyloid beta_1–42_ and neurofibrillary tangles, were found in brain of aged cynomolgus monkey. The aim of this study was to investigate if aged monkeys exhibiting spatial memory impairment and levels of biomarkers indicative of AD, had brain lesions similar to human patients suffering from senile dementia. Generating immunohistochemistry technique to biomarkers of amyloid beta_1–42_ and the phosphorylated tau 231, our study assessed the amyloidopathy, such as indicative to the senile plaques and cerebral amyloid angiopathy, and the tauopathy, to possible neurofibrillary tangles. Six aged monkeys were selected based on their spatial memory performance and profile of biomarkers of AD, divided equally to affected aged subject – with Memory-affected and low amyloid level, and aged with higher performance in memory and amyloid, as the age-matched subjects. Using immunohistochemistry, plaques of amyloid beta_1–42_ were observed in two out of three brains of aged subjects with memory impairment and biomarkers indicative of AD. The cerebral amyloid angiopathy was observed in both aged monkey groups, and unlike in the human, the amyloids were found to deposit in the small veins and capillaries. In one of the affected individuals, phosphorylated tau was positively stained intracellularly of the neurons, indicating a possibility of an early stage of the formation of tangles. These findings add to the body of evidence of the utility of the aged cynomolgus monkeys as a spontaneous model for Alzheimer-related disease.

## Introduction

Tests for several behavioral tasks, developed within the human neuropsychological domain, have been successfully adapted for use with non-human primates (NHP), including delayed response tasks (DRT) (Amici et al., 2010) where delays of various lengths are imposed between the presentation of a stimulus and the desired response (Bartus and Dean, 2009; Lacreuse and Herndon, 2009; Rodriguez and Paule, 2009; Nagahara et al., 2010). This type of memory tests is appropriate in assessing Alzheimer’s disease (AD), and as well as other affected cognitive function such as executive function and divided attention (Johannsen et al., 1999; Giannakopoulos et al., 2007). As some of the earliest cerebral lesions in the AD progression are typically located in the hippocampus (Deiana et al., 2011), spatial memory tests are of particular interest.

Delayed response tasks performance in cynomolgus monkeys (*Macaca fascicularis*) has recently been described (Darusman et al., 2013a) demonstrating that old monkeys (more than 20 years of age) performed poorer than young (4–9 years) and middle aged (10–16 years) individuals. The DRT performance was correlated with the levels of the core biomarkers indicative of AD, especially the amyloid-beta_1–42_ (Aβ_42_) (Darusman et al., 2013b, 2014), where the DRT performance was positively correlated with concentrations of Aβ_42_. Structural magnetic resonance imaging (MRI) studies identified abnormalities, such as atrophy in hippocampus and morphological changes in the cortical areas in aged monkeys with poor memory and low Aβ_42_ levels (Darusman et al., 2014).

Earlier studies of brain sections from older cynomolgus monkey revealed pathological hallmarks indicative of AD (Nakamura et al., 1998), and based on the combined findings described above, the present study was carried out to investigate whether animals with poor DRT performance and circulating biomarkers levels indicative of senile dementia also exhibited brain lesions such as senile plaques (SP) of Aβ_42_ and tauopathy. In the human, the progression of cognitive decline and biomarkers levels reflect the AD pathological processes in the brain (Andreasen and Blennow, 2005; Perrin et al., 2009). Low levels of circulating Aβ_42_ and elevated total tau (t-tau) and phosphorylated tau (p-tau), are related with the development of SP and NFT, respectively (Brody et al., 2008; Blennow et al., 2010; Jack et al., 2010; Albert et al., 2011). In rhesus monkeys, the incidence, distribution, and chemical composition of the Aβ deposits in the brain of young and aged individuals have been described (Sani et al., 2003; Nishimura et al., 2012) with higher level of Aβ_1–40_ (Aβ_40_) than the Aβ_42_.

In the present study, we examined the presence of Aβ_42_ and the p-tau by immunohistochemistry analysis in brain sections of aged monkeys selected from a previous study (Darusman et al., 2013a,b), based on low total DRT and low levels of Aβ_42_ in cerebrospinal fluid (CSF) compared with aged monkeys with better memory performance and high CSF Aβ_42_ levels. The DRT were assessed by the short term memory test (STMT), long term memory test (LTMT), and memory load test (MLT).

## Materials and Methods

### Subjects

From previous studies, we selected aged subjects above 20 years old that met the requirements of low levels of Aβ_42_(<5000 pg/ml) and low DRT performance (<40%) for the memory-affected group (Darusman et al., 2013a,b). Aged subjects characterized by high-circulating Aβ_42_ and high DRT performance were selected for the age-matched control group. Subject age was determined from birth certificates for the animals born in captivity and from dental scaling (Swindler, 2002) and estimated year of born for animals born in the wild.

The subjects in this study (Table 1) were limited to old individuals that were destined for euthanasia due to progressive weight loss, paleness of the mucous membranes, reduced appetite, and/or general weakness. These criteria yielded three memory-affected monkeys (two females and one male), and three age-matched monkeys (one female and two males). Two young monkeys (one female and one male) were included to control for potential confounds associated with age.

**Table 1:** **The characteristics of the subjects**.

Tattoo/sex/age group	Total DRT (%)	Biomarker (pg/ml)			Reference	Age^a^
		Aβ_42_	t-tau	pT231	
C2538/female/young	68.09	655.10	301.60	2.36	Darusman et al. (2013a)	9
C0744/male/young	60.50	620.00	568.60	6.27	Darusman et al. (2013a)	7
10063/female/memory-affected	40.58	16.01	92.62	4.81	Darusman et al. (2013b)	30
T3311/male/memory-affected	40.00	368.36	319.46	4.19	Darusman et al. (2013a)	30^b^
I1112/female/memory-affected	34.34	164.96	219.85	5.45	Darusman et al. (2013a)	29
10749/female/age-matched	61.67	416.36	370.57	4.11	Darusman et al. (2013b)	30
9661/male/age-matched	60.16	480.76	277.71	7.55	Darusman et al. (2013b)	27
T3283/male/age-matched	66.42	524.65	60.64	4.37	Darusman et al. (2013b)	30^b^

*Data are collected from the references Darusman et al. (2013a,b). DRT performance is presented as the collected percent of trials where the subject could correctly retrieve a hidden item following a predetermined delay. Biomarker levels were measured in serum and CSF*.

*^a^Up to the end of year of 2013*.

*^b^Estimated from the year of birth by a population survey in 1983*.

All subjects were housed at the AAALAC-accredited Primate Research Center, Bogor Agricultural University (PRC, IPB) in pairs or social groups of various sizes. During the testing period, the subjects were individually housed indoors in adjacent cages, which permitted some tactile contact through perforated acrylic panels. The subjects’ housing conditions and test procedures were approved by the PRC IPB Animal Care and Use Committee. The subjects’ characteristics with their previous DRT performance and biomarker levels are presented in Table 1.

### Histopathology

The animals were euthanized with pentobarbital and phenytoin injections (Euthasol^™^, Virbac, Fort Worth, TX, USA) followed by intracardial sodium chloride 0.9% perfusions and exsanguination; the intracardial perfusion was continued with paraformaldehyde (4%). Brains were collected and immersed in 10% paraformaldehyde solution for 24 h. Subsequently, the brains were fixated in buffered formalin 10% (pH 7). Fixated brains were sectioned in the coronal plane allowing for analyses of five distinct regions: the frontal, occipital, parietal and temporal lobes, and the hippocampus.

All sections were embedded in paraffin wax and cut into 5 μm sections. Hematoxylin-eosin (HE) staining was applied for routine examination of the brain, especially for observing degeneration signs (apoptotic neuron signs such as chromatolysis, gliosis, vacuolization) because we were not applying immunohistochemistry studies of these degeneration signs. Congo-red staining was also applied to observe amyloid in general. For immunohistochemistry of Aβ_42_ and p-tau, sections were treated with sodium citrate buffer at 95°C for 30 min, and to minimize non-specific binding, sections were treated with 3% hydrogen peroxide in methanol for 30 min at room temperature, and blocked using 1% fetal bovine serum and 10% skimmed milk in PBS for 30 min at room temperature. An AD detection kit (Millipore™, Temecula, CA, USA) was used to identify Aβ_42_ and p-tau threonine 231 (pT231). The kit contained polyclonal antibodies against human Aβ_42_ (catalog number AB5078P) and pT231 (catalog number MAB3420SP).

The sections were incubated with the specific antibodies (diluted 1:500 in PBS) overnight at 4°C. After washing with PBS, the sections were incubated with a biotinylated secondary antibody for 30 min, and subsequently with Streptavidin-Horse Radish Peroxidase (HRP) for 30 min at room temperature (DAKO-LSAB^+^ system HRP kit; Glostrup, Denmark). Diaminobenzidine (DAB) was applied at room temperature until an appropriate intensity was obtained (2–5 min). Sections were rinsed in distilled water, counterstained with HE to visualize tissue morphology and then mounted with Entellan^®^ (product number 1079610100, Merck KGaA, Darmstadt, Germany). Negative controls (without secondary antibody), antibody control (treated with normal rabbit serum), and photomicrograph control, were included. All slides (three slides for each brain region, times each staining procedure were analyzed for each subject; i.e., 3 × 5 × 7 = 105) were scored independently by two trained pathologists that were blinded to the identity of the individuals. Scores are summarized in Table 2.

**Table 2:** **Scoring criteria of the histopathology of the brain**.

Score	Degeneration	Amyloid disorders		Tauopathy
		CAA	Senile plaques	
1	Condensed chromatin inside nuclei (chromatolysis)	Deposit of amyloid in vascular wall of arterioles	Diffused plaques – appearing as small numbers of nodules in the brain parenchyma	p-tau detected inside the cytoplasm of the neuron (intracellular stage)
2	Aggregation of satellite cell/glial cell (gliosis)	Deposit of amyloid in vascular wall of venules	Compacted plaques – appearing in larger amounts and sizes in the brain parenchyma	p-tau detected in the cytoplasm and/or axon of the neuron (fibrillar stage)
3	Formation of vacuole sites as indicative of active phagocytosis of the degenerated neuron (vacuolization)	Deposit of amyloid in vascular wall of capillaries	Compacted plaques in the brain parenchyma associated with degeneration of the surrounding nerve	p-tau detected as tangled formations outside of the neuron (extracellular stage)

The degeneration score follows the progression of the apoptotic nerve cell (Kettemann et al., 2011). The degree of chromatolysis and the number of satellite cells or glial cells (Gliosis) was also incorporated in the degeneration score (Chen et al., 2013). Two aspects of amyloidopathy were scored: cerebral amyloid angiopathy (CAA) and SP (Heuer et al., 2012), where CAA scores were based on the localization of Aβ_42_ (Attems and Jellinger, 2004) and plaques were scored by morphology (lowest score was valued when the plaques appeared as small numbers and diffused, continued with compacted formation and compacted plaques with sign of degeneration surrounding as the highest score). The tauopathy was scored based on which stage the tangles formation was perceived to be in Augustinack et al. (2002).

## Results

### Degeneration

The histopathology findings (scores and the lobes where the lesion appeared) are presented in Table 3. Signs of brain degeneration were found in the aged monkeys, both the memory-affected and aged-matched subjects. Degeneration signs such as gliosis and vacuolization were present in the brains of all aged, but not in young animals. Early signs of degeneration were found in young animals, observed as a chromatolysis, while the later stages – the gliosis and vacuolization – were only observed in aged animals, both the memory-affected and age-matched subjects. These degeneration signs were observed in the frontal lobe of all the aged subjects (*n* = 6), in the parietal lobe of most (*n* = 5), and in the temporal and occipital lobes of some (*n* = 2). A degeneration of the hippocampus was found only in the memory-affected aged monkeys observed as chromatolysis.

**Table 3:** **Histopathology findings and summary of the scores**.

Tattoo	Group	Average score from two observers (lobes^a^)			
		Degeneration	CAA	Senile plaques	Tauopathy
C2538	Young	1(P)	0	0	0
C0744	Young	1(P)	0	0	0
10063	Memory-affected	2(F), 2(P), 1(O), 1(Hip)	3(F), 2(T)	0	0
T3311	Memory-affected	3(F), 2(T), 1(Hip)	3(F), 2(P), 1(O)	2(F), 1(P), 1(Hip)	0
I1112	Memory-affected	3(F), 2(T), 1(O), 1(P), 1 (Hip)	2(F), 2(T), 1(O), 1(P), 1 (Hip)	2(F), 1(T), 1(O), 1(Hip)	1 (T), 1 (O)
10749	Age-matched	3(F), 2(P)	2 (F), 2(T), 1(Hip)	0	0
9661	Age-matched	3 (F), 2 (P)	2 (F), 1(T), 1(P)	0	0
T3283	Age-matched	2(F), 2 (P), 1(O)	2(F), 2 (T), 1 (O)	0	0

*^a^F, frontal lobe; T, temporal lobe; P, parietal lobe; O, Occipital lobe; Hip, hippocampus*.

### CAA

**Figure 1:**
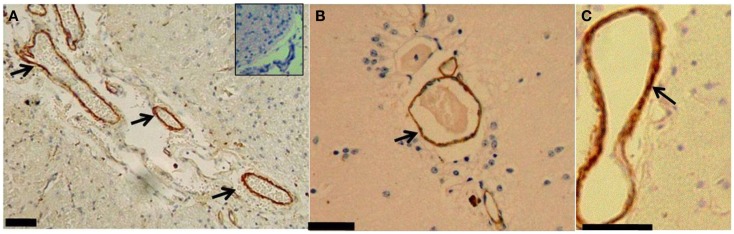
**(A)** Brain section from the temporal lobe of a memory-affected animal (subject T3311) treated with a polyclonal serum against human Aβ42 and stained with DAB. The inset picture is a photomicrograph control treated with nonsense rabbit serum. **(B)** The occipital lobe of an Aged-matched subject (subject 10749); and **(C)** the hippocampus (subject I1112); arrows indicate deposition of the Aβ_42_ inside the walls of small veins. Scale bars: 60 μm.

The CAA was found in the small vessels of the brain in all of the aged monkeys. Brown coloration from DAB appeared in the vascular wall of the brain capillaries, preferentially in small veins (Figure 1). The CAA was observed in the blood vessels of the frontal lobe (*n* = 6), temporal lobes (*n* = 5), occipital and parietal lobes (each *n* = 3), and hippocampus (*n* = 2). The CAA was observed in the capillaries of the frontal lobe in the Memory-affected monkeys (*n* = 2).

### Senile plaques

**Figure 2:**
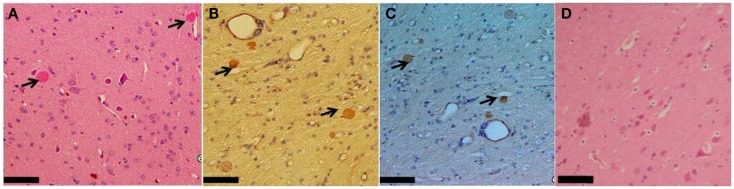
**Brain section of the frontal lobe from a Memory-affected animal (subject I1112) demonstrating Aβ_42_ plaques in the parenchyma, (A) stained with HE, (B) Congo red and (C) with anti-Aβ_42_ antiserum/DAB, and (D) HE from Aged-matched subject (subject 10749)**. Arrows indicate Aβ_42_ plaques. Scale bars: 60 μm.

Plaques of Aβ_42_ in the parenchyma were observed in the brain from two monkeys of the memory-affected group (Figure 2); subjects I1112 and T3311. The plaques were principally found in the frontal, temporal and parietal lobes, and the hippocampus. The compacted shape of the plaques was observed only in frontal lobes, while the diffused shapes were found in the other aforementioned lobes.

### Tauopathy

**Figure 3:**
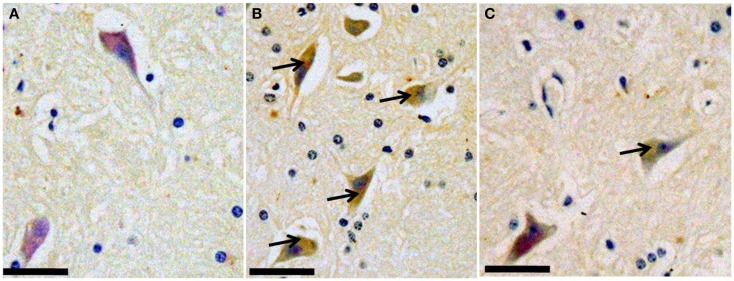
**The occipital lobe of an age-matched subject (T3283) (A), the occipital lobe of a memory-affected subject (I1112) (B), and temporal lobe (C)**. Sections have been treated with a polyclonal serum against human p-tau pT231 and stained with DAB. Arrows indicate the presence of pT231 in the cytoplasm of nerve cells. Scale bars: 60 μm.

The pT231 was found in the temporal and occipital lobe from one subject belonging to the memory-affected group (I1112). The pT231-positive structures appeared only in the cytoplasm of the neuron cell (Figure 3). The brownish colorization from the pT231 antibody was weaker compared with the other DAB staining of the SP and CAA.

## Discussion

In addition to degenerative lesions, such as the microgliosis and vacuolization, we found the CAA in both aged groups and not in the young individuals – defined by an accumulation of amyloid beta in the brain vascular wall: cerebral beta-amyloid angiopathy (Rensink et al., 2003; Revesz et al., 2003, 2009; Greenberg et al., 2004). Since both aged groups developed the CAA, it suggested that the CAA may occur spontaneously in cynomolgus monkeys as in other aged NHP species, such as Rhesus monkeys (Walker, 1997; Heuer et al., 2012).

Qualitatively, the CAA in the memory-affected and aged-matched groups shared same relative amounts and similar to location in lobes where the lesions were present, namely the frontal and parietal-temporal lobes, which are the brain lobes related to memory function and also affected in AD and frontotemporal disease (FTD) in the human (Naggara et al., 2006; Rodriguez and Paule, 2009; Heuer et al., 2012), along with the hippocampus area (Johannsen et al., 2000; Deiana et al., 2011). CAA is present in almost in all cases of AD (Okamoto et al., 2009, 2012).

In humans, CAA is most prominent in the arterioles and less frequent in veins and capillaries (Preston et al., 2003), while interestingly our findings showed that amyloid beta deposits in the small veins and capillaries of the cynomolgus monkey. More thorough study of the mechanism of Aβ deposition in the veins is required in order to confirm whether or not the Aβ internalizes into endothelial cells, from blood passing through capillary pores, and thereby becomes detectable inside the vessel wall of veins and capillaries. The suspected CAA inside the capillaries and small veins correlates with AD pathology (Attems and Jellinger, 2004) and cognitive impairment (Eurelings et al., 2010). Our findings thus support studies of CAA in other aged old world monkeys (Walker, 1997; Nakamura et al., 1998; Oikawa et al., 2010).

In rhesus monkey and cynomolgus monkey, CAA and the parenchymal plaques of amyloid beta, are correlated (Nakamura et al., 1998; Levine and Walker, 2006) and our findings may support this. Our findings suggest that memory impaired cynomolgus monkeys suffer from an amyloid-related disease manifested as SP in the brain. On the other hand, animals with spontaneous amyloid-like disorder such as the dog with canine cognitive dysfunction had a fundamentally different relationship between the cognitive disorder and the retention of amyloid load sensitive marker Pittsburgh compound B (Fast et al., 2013).

Among three Memory-affected subjects, we found two subjects (I1112 and T3311) with the parenchymal form of amyloid beta inside the frontal, temporal lobes, and the hippocampus. The plaques were present in the same lobes where CAA was found. In the rhesus monkey, the deposits of Aβ were observed principally in the cortical, paralimbic, and core limbic cortical zones corresponding to mild, moderate, and heavy burden, respectively (Sani et al., 2003). Along with Nishimura et al. (2012), this study of rhesus monkeys also emphasizes the higher proportion of Aβ_40_ compared with the Aβ_42_. The absence of Aβ_40_ analysis in our study seems limit the whole interpretation.

However, another study in the species showed that the Aβ42 often predominate the Aβ40 in the cortical level of both temporal and occipital lobes, although the relative level of Aβ40 remain higher in the occipital cortex (Rosen et al., 2011). The additional immunohistochemistry of Aβ_40_ could add more information about the proportion of the Aβ in the cynomolgus monkey. This would support a biomarker study by Yue et al. (2014), which describes that Aβ_42_ was significantly associated with aging but not with the Aβ_40_ in cynomolgus monkey.

Similar to other studies (Oikawa et al., 2010; Heuer et al., 2012), we also found signs of both nerve damage and tangle formation in the brain of aged monkeys although the location of the plaques and the tangles were not correspondently the same. An old female from the memory-affected group (I1112), which had the parenchymal form of amyloid beta stained positively for pT231 in the body of nerve cells in the temporal and occipital lobes. In AD patients, this is the early stage of NFT, indicated by the intracellular form of NFT (Augustinack et al., 2002). The identified p-tau in the neuron’s cytoplasm was not corresponded with the SP, showed by the differences of the lobes’ location between SP and p-tau. This finding agrees with a previous study in cynomolgus monkey (Oikawa et al., 2010) that the pathological differences of SP and tauopathy in the species is specific and may differed with human’s AD pathology.

Although the present study was small, it adds to the body of evidence (Oikawa et al., 2010; Heuer et al., 2012) that support the neuropathology of the aged cynomolgus monkey as a potential model for studies of spontaneous disease of Alzheimer-type, especially the amyloid-related brain pathology and also suggests the potential for further study of p-tau mediated pathology in the species (Walker, 1997; Levine and Walker, 2006; Oikawa et al., 2010; Heuer et al., 2012). Future studies of high relevance would include more detailed analyses of the Aβ_40_, Aβ_42_, and the pT231 presence in each of the brain lobes to confirm the expression of both biomarkers and to complement the histopathology results.

## Conflict of Interest Statement

The authors declare that the research was conducted in the absence of any commercial or financial relationships that could be construed as a potential conflict of interest.
